# The Interplay of Reactive Oxygen Species, Hypoxia, Inflammation, and Sirtuins in Cancer Initiation and Progression

**DOI:** 10.1155/2016/3907147

**Published:** 2015-12-20

**Authors:** Marco Tafani, Luigi Sansone, Federica Limana, Tania Arcangeli, Elena De Santis, Milena Polese, Massimo Fini, Matteo A. Russo

**Affiliations:** ^1^Department of Experimental Medicine, Sapienza University of Rome, 00161 Rome, Italy; ^2^Department of Cellular and Molecular Pathology, IRCCS San Raffaele, 00166 Rome, Italy; ^3^Consortium MEBIC, San Raffaele University, 00166 Rome, Italy; ^4^Department of Gynecological-Obstetrical Sciences and Urological Sciences, Sapienza University of Rome, 00161 Rome, Italy; ^5^Department of Human Anatomy, Sapienza University of Rome, 00161 Rome, Italy

## Abstract

The presence of ROS is a constant feature in living cells metabolizing O_2_. ROS concentration and compartmentation determine their physiological or pathological effects. ROS overproduction is a feature of cancer cells and plays several roles during the natural history of malignant tumor. ROS continuously contribute to each step of cancerogenesis, from the *initiation* to the *malignant progression*, acting directly or indirectly. In this review, we will (a) underline the role of ROS in the pathway leading a normal cell to tumor transformation and progression, (b) define the multiple roles of ROS during the natural history of a tumor, (c) conciliate many conflicting data about harmful or beneficial effects of ROS, (d) rethink the importance of oncogene and tumor suppressor gene mutations in relation to the malignant progression, and (e) collocate all the cancer hallmarks in a mechanistic sequence which could represent a “physiological” response to the initial growth of a transformed stem/pluripotent cell, defining also the role of ROS in each hallmark. We will provide a simplified sketch about the relationships between ROS and cancer. The attention will be focused on the contribution of ROS to the signaling of HIF, NF*κ*B, and Sirtuins as a leitmotif of cancer initiation and progression.

## 1. Introduction

ROS (Reactive Oxygen Species) production has been strictly associated with cancer [1], ageing [2], diabetes [3], obesity [4], neurodegeneration [5], and other age-related diseases such as age-related retinopathy, cochlear degeneration, and chronic inflammatory diseases [6]. How can ROS contribute to so many apparently different clinical entities and what are the common molecular targets and pathways altered by ROS? In recent years, a great amount of information has been produced to answer these questions. Interestingly, such information stems from the study of the roles of ROS along the tumorigenesis sequence [7].

The complexity of relationships between ROS and cancer pathogenesis is primarily due to the diverse species of ROS produced by O_2_ metabolism and their properties, such as chemical nature, half-life, reactivity and specificity for their biological targets, ability to diffuse and travel among subcellular compartments, type of changes produced in target molecules, and, finally, the importance of affected biological functions [8]. Moreover, it is difficult to identify the molecular targets and the numerous redundant pathways modified by ROS, with a significant role in cancerogenesis. Besides, biologically active or toxic concentrations of ROS resulting from the ratio between production and detoxification introduce additional important variables to be considered in describing the ROS/cancer relationships [9].

Cancer pathogenesis may be described as a multistep process including* transformation*,* growth promotion *and, in clinically evident tumors,* malignant progression *[10]. During the natural history of cancer a large number of genes, molecules, and pathways contribute first to transformation and promotion then to the manifestation of the malignant cancer phenotype; most of these molecules and pathways interact with ROS in the cytosol, nucleoplasm, and intraorganellar space.

A transformed cell is identified by the loss of control of proliferation and deregulation of apoptosis producing an excess of cell number and forming a mass (tumor). The disruption of cell cycle and apoptosis regulation is due to mutations of genes with a gain-of-function (oncogenes) and a loss-of-function (oncosuppressor genes), both leading to an excessive proliferative signal [11, 12]. The deregulation of apoptosis is due to mutations of genes involved in the signaling controlling programmed cell death, with a gain-of-function of genes (oncogenes) protecting from apoptosis and a loss-of-function (oncosuppressor genes) promoting apoptosis. Upstream, alterations of DNA repair mechanisms may often facilitate the accumulation of crucial mutations in a single stem cell giving rise to the transformed stem cell responsible for the growth of the early small tumor [13].

The initial growth of a small tumor occurs with absent, insufficient, or abnormal angiogenesis. This produces areas of hypoxia of different severity in which ROS increases, favoring tumor cell survival, adaptation, and progression [14]. Even though the precise mechanism through which hypoxia increases ROS is still a matter of debate, it seems that ROS production is due to the effects of hypoxia on the mitochondria electron transport chain (ETC). In particular, hypoxia would drive ROS increase by acting on complexes I, II, and III of the ETC [15, 16]. In fact, the use of inhibitors for each one of these complexes resulted in the inhibition of ROS accumulation [15, 16]. Moreover, such ROS are mainly represented by H_2_O_2_ since forced expression of catalase or glutathione peroxidase-1 completely reversed hypoxia-induced ROS expression in isolated pulmonary artery myocytes [15, 16]. Interestingly, hypoxia-driven ROS increase would then leave the mitochondria causing destabilization of Prolyl Hydroxylases (PHD) and stabilization of HIF1*α* [15, 16].

HIF is the major transcription factor responsible for triggering tumor progression [17]. In addition, in this phase, ROS further increases contributing to the involvement of NF*κ*B and Sirtuins in the full acquisition of malignant phenotype [18].

Here we will shortly review the contributions and mechanisms of ROS from cell transformation to the acquisition of every single hallmark of a clinically significant malignant tumor, trying to correlate specific molecular targets to ROS role.

## 2. ROS Compartmentation and Production

**Figure 1:**
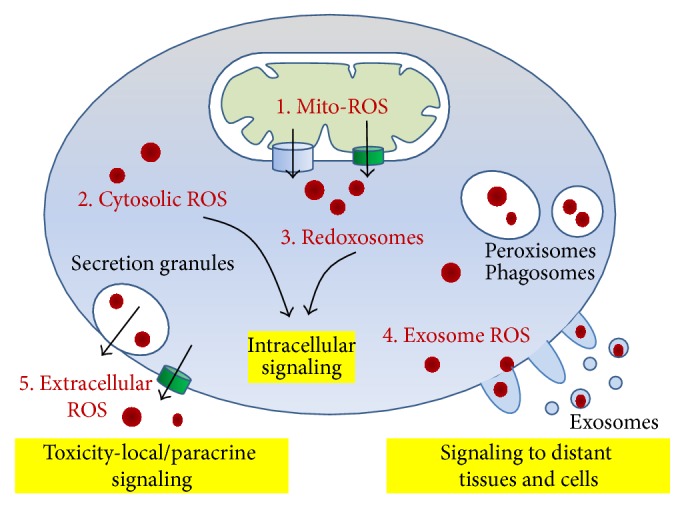
Subcellular compartmentation of ROS. 1. Mitochondrial ROS which can travel to cytoplasm through VDAC (superoxide) or through aquaporin (peroxides). 2. Cytosolic ROS. 3. Redoxosomes, such as peroxisomes and endoplasmic reticulum derived vesicles. 4. ROS included into exosomes and vesicles shedding from damaged plasma membranes. 5. Extracellular ROS in extracellular fluids and plasma, partly crossing the plasma membrane through aquaporin, partly secreted with granules (i.e., activated leukocytes).

Five main compartments contain ROS: mitochondria, cytosol, single membrane-bound organelles (peroxisomes, endosomes, and phagosomes), exosomes released by plasma membranes by shedding, and extracellular fluids including plasma [9]. As schematized in Table 1 and Figure 1, ROS are produced in different subcellular compartments by the action of different enzymes and then they can travel through channels or vesicles. In particular, mitochondria produce large amount of ROS that can be either detoxified or can leave the organelle through channels such as voltage dependent anion channel (VDAC) or aquaporin. Similarly ROS can be produced by NADPH-oxidases (NOX) and other cytosolic enzymes as well as by peroxisomes. Finally, ROS can be released in the extracellular space through aquaporin or exosomes (Figure 1).

Three broad classes of ROS may be produced: hydroxyl radicals, superoxides, and hydroperoxides, with distinctive characteristics regarding their reactivity, half-life, target specificity, localization, and, very importantly, biological and pathological effects (Table 1). At present, the acronym ROS may include also several nitrogen-containing compounds or RNS (Reactive Nitrogen Species), such as nitric oxide (NO), nitroxyl anion (NO^−^), and peroxynitrite (ONOO^−^). NO is produced by the activity of inducible nitric oxide synthase (iNOS) and reacts with superoxide to give rise to the other RNS. ROI (Reactive Oxygen Intermediates) and RNI (Reactive Nitrous Intermediate) are additional acronyms used to indicate ROS [8, 19].

**Figure 2:**
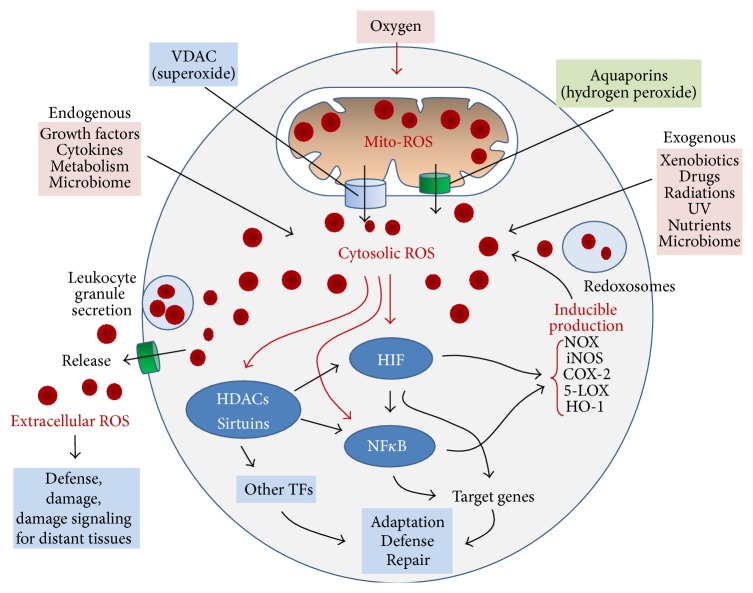
ROS are produced mainly in the mitochondria. Superoxides are rapidly detoxified by mitochondrial MnSOD as hydrogen peroxide or can cross mitochondrial membranes through the VDAC. Hydroperoxides travel easily to cytosol through membrane aquaporin. In addition to ROS coming from mitochondria, cytosolic ROS can originate from many endogenous or exogenous sources, including nutrients, radiation, microbiome, growth factors, cytokines, and other metabolisms. Proinflammatory inducible enzymes such as NADPH-oxidases (NOX), inducible nitric oxide synthase (iNOS), inducible cyclooxygenase (COX2), 5-lipoxigenase, and inducible heme-oxigenase-1 (HO-1) may produce an additional burst of ROS. HIF1*α*, NF*κ*B, and HDACs, especially Sirtuins, are activated by ROS in synergy with the specific signaling from receptors and metabolism. Target genes of activated TFs are aimed at adaptation to hypoxia, proinflammatory harmful agents' inactivation, and damage repair. ROS are also released in the extracellular space by secretion of granules of activated leukocytes or crossing plasma membrane through anionic channels (superoxides) or aquaporins (hydroperoxides). Extracellular ROS are important for defense (as in case of ROS released by eosinophils against macroparasite) and produce collateral damage not only in adjacent healthy tissues but also in distant tissues and organs, signaling the local damage and activating improper mechanisms of adaptation, remodeling, and chronic damage.

ROS are produced in the* mitochondria* as by-products of fatty acid (FA) metabolism and oxidative phosphorylation for ATP synthesis [8, 19].* Hydroxyl anion* half-life is extremely short (10^−9 ^sec) interacting with and sometimes damaging any biological molecule in its range.* Superoxides *encounter two destinies: rapid detoxification by mitochondrial MnSOD (Mn-dependent superoxide dismutase) as hydrogen peroxide or mitochondrial membrane crossing through the VDAC.* Hydroperoxides* travel easily to cytosol through membrane aquaporin [8] (Figure 2).

*Cytosol* can produce ROS from many endogenous (growth factors, cytokines, and metabolisms) or exogenous sources (nutrients, radiation, microbiome, and xenobiotics). On the other hand, cytosol can accumulate ROS produced by mitochondria and redoxosomes, especially superoxide and hydroperoxides. ROS and RNI, accumulating into the cytosol, can diffuse easily (depending on half-life) into the nucleoplasm, interacting with nucleic acids and other nuclear components [20].

In the cytosol and redoxosomes, proinflammatory inducible enzymes such as NADPH-oxidases (NOX), inducible nitric oxide synthase (iNOS), inducible cyclooxygenase (COX2), inducible 5-lipoxygenase (5-LOX), and inducible heme-oxygenase-1 (HO-1) may produce an additional burst of ROS.

In particular, the different isoforms of NOX, identified in many tissues and cells, are an important source of ROS in response to different stimuli including hypoxia [21]. NOX is a multisubunit enzyme complex generating superoxide by one-electron reduction of oxygen using reduced NADPH as the electron donor [21]. NOX is widely distributed among different species, suggesting that such enzyme plays an important role in the cell. However, the precise physiological role of NOX is still unclear, whereas its pathophysiological role is definitely lined to ROS production and ROS-induced damage [22]. Finally, as for HIF (see below), mitochondrial ROS accumulation following hypoxia can, in turn, activate NOX through a mechanism requiring protein kinase C*ε* and leading to further ROS increase and cellular damage [23].

Hypoxia-induced ROS accumulation also increases expression and activity of 5-LOX in pulmonary artery endothelial cells with production of leukotrienes and induction of cell proliferation [24].

The presence of the cytosolic CuSOD (Cu-dependent superoxide dismutase) and of a number of scavenging molecules, that is, peroxiredoxins and glutathione peroxidase, [22, 25, 26] detoxifies the excess of cytosolic ROS (Figure 2).

A special case of ROS production and utilization occurs in the* redoxosomes*. A number of oxidases are localized in specialized stable (peroxisomes) or transient (phagosomes, multivesicular bodies, endosomes, etc.) single-membrane bound organelles that can produce substantial amount of ROS as typically occurs in the respiratory burst of activated leukocytes (macrophages and eosinophils) or during peroxisome proliferation in response to xenobiotics [27].

Members of NOX family (NADPH-oxidases) and myeloperoxidase are induced and confined in the vacuole microenvironment where they produce a large amount of ROS mainly aimed at killing bacteria and inactivating harmful substances. This represents an efficient defense mechanism against bacteria and parasites [28].

Variable ROS concentrations have been measured in many extracellular fluids, such as blood plasma and spermatic, peritoneal, and pleural fluid [29, 30]. Free* extracellular ROS* have two origins: from cytosol crossing the plasma membrane through aquaporins (hydroperoxides) and some anion channels (superoxides) and by secretion (external opening of phagosomes and granules) as typically occurs in activated degranulating leukocytes [31, 32]. The range of action of extracellular ROS is determined by their half-life, reactivity, velocity of diffusion, and the possibility to travel with plasma. More reactive and short-living ROS (hydroxyl anion and superoxide) act in a short range damaging local biological structures (i.e., macroparasites and adjacent tissue cells), while hydroperoxides may travel with plasma contributing to determining the redox levels of the blood and thus influencing the activity and the life of blood cells and of important plasma proteins [33, 34]. ROS are also released in the extracellular space by secretion of granules of activated leukocytes or crossing plasma membrane through chloride and other anion channels (superoxides) and aquaporins (hydroperoxides) [35]. Extracellular ROS are important for defense (as in case of ROS released by eosinophils against macroparasite) and produce collateral damage not only in adjacent healthy tissues but also in distant tissues and organs, signaling the local damage and activating improper mechanisms of adaptation, organ remodeling, and chronic damage (Figure 2).

Recent literature has recognized the functional importance of* exosomes*. Exosomes are small (50–90 nm) vesicles originating from invagination of multivesicular bodies and plasma membrane and are released in small amount by normal cells and in large number by cell under various types of stress and by cancer cells, diffusing and traveling through extracellular biological fluids [36, 37]. Their content is largely determined by the local cytosolic composition where exosomes are formed and therefore, their content, includes water, ions, soluble metabolites proteins nucleic acid, and ROS. Their membrane contains membrane-associated proteins including ligands which can allow exosomes to interact with distant cells and tissues expressing the corresponding receptor. After this interaction external and internal molecules can enter target cells initiating signal cascades that can influence cell physiology and pathology. In particular, exosomal microRNA and proteins have been demonstrated to play a role in distant organ remodeling and damage developing multiorgan diseases as observed in complex patients [37].

## 3. ROS Biological Functions and Damaging Effects

The increase of ROS following hypoxia has been extensively documented using different techniques. However, precise numbers indicating the level of ROS generation in tumors are difficult to obtain due to the multiple antioxidant pathways and molecular mechanisms activated by tumors to survive to such an increase and to thrive. An important aspect is the interaction of ROS with different cellular components that produces different types of changes depending on the classes of ROS. In particular, as shown in Table 1, hydroxyl radicals are highly reactive causing sublethal or lethal degradation (toxicity), whereas superoxide and hydrogen peroxide have a lower reactivity but can cause local damage or activation of signaling cascade when present at physiological concentration. Lipids, proteins, and nucleic acids (sugar backbone and N-bases) are the most significant targets of ROS-induced damage [38].

Chronic oxidative stress exerts detrimental effects during the multistage process of carcinogenesis, including DNA damage, impaired DNA repair, mutations in tumor suppressor genes, epigenetic changes, altered apoptosis, disruption of signal transduction pathways responsible for maintaining the normal cellular homeostasis, angiogenesis, and metastasis. For a comprehensive description of ROS-induced DNA damage we refer the reader to the reviews of Ziech et al. and Caputo et al. [39, 40].

*Lipoperoxidation* has the most significant impact on plasma membrane structure and permeability. Plasma membrane damage disrupts ionic gradients: the entry of Na^+^ and water leads to cell swelling (one of the most frequent cell alterations in mammalian tissue pathology). However, the necrotic catastrophe is associated with the entry of extracellular Ca^++^. Disruption of Ca^++^ homeostasis leads to a rapid cell degradation through (1) a further increase of ROS production and damage [41], (2) an abnormal function of cytoskeletal components (supercontracture) [42, 43], and (3) an abnormal activation of Ca^++^-dependent proteases, such as calpains, caspases, and proteasomes [44]. To this effect it is important to consider that Ca^++^ homeostasis maintenance depends on the cellular compartment roughly as follows: (i) cytosolic ([pCa_i_^++^] = 10^−9 ^M); (ii) endoplasmic reticulum cisternae ([pCa^++^] = 10^−6 ^M); (iii) mitochondrial ([pCa_m_^++^] = 10^−5 ^M); (iv) extracellular ([pCa_e_^++^] = 10^−3 ^M).

*Superoxides* react rapidly with iron-sulphur groups of proteins or with NO generating peroxynitrite, which, in turn, acts on proteins (tyrosine nitration and S-glutathionylation) [45]. Hydroperoxides act by oxidizing cysteine residues in proteins and influencing deeply their activity. Mitochondrial DNA and nuclear DNA undergo several alterations that may result in mutation accumulation and genomic instability [46]. Mitochondria undergo mtDNA alterations and metabolic dysfunction with increase in ROS production. Nuclear DNA undergoes point mutations, breaks and consequent deletion, inversion, and translocation, all conditions that can activate oncogenes or inactivate oncosuppressor genes.

Several proteins, targets of ROS, play a crucial role during tumor progression. Herewith we will focus the attention on HIF, NF*κ*B, and Sirtuins. It is interesting to note that a full activation of HIF, NF*κ*B, and Sirtuins occurs in synergy with specific signaling from receptors or other pathways following a previous interaction with ROS.

### 3.1. ROS May Induce Cell Transformation through Mutations

ROS are at the early origin of cancer. Radiations, UV, xenobiotics, chemical carcinogens, nutrients, and chronic inflammation are sources of mitochondrial and cytosolic ROS. In the nucleus they damage in different ways DNA producing random mutations including those that allow a normal cell to lose the control of cell cycle and of the apoptosis [47]. Most of the times, mutations are corrected by one of the DNA repair mechanisms such as double strand break (DSB) repair, base excision repair (BER), mismatch repair (MMR), and, possibly, nucleotide excision repair (NER) [48]. Alternatively, the cell can undergo apoptosis. In these conditions, the chances to select a transforming combination of mutations are substantially increased by defective DNA repair mechanisms, by predisposing germline mutations and by defective ROS detoxifying systems. In conclusion, ROS mediate the mutagenic action of a number of carcinogenetic agents playing a prevalent role in the initial transformation of a normal cell into a tumor cell.

### 3.2. ROS Promote Growth and Genomic Instability in Already Transformed Cells

A second contribution to cancerogenesis is given by additional ROS constitutively produced in transformed cells by mutated oncogenes. In particular, oncogenes such as Ras and Myc, often overexpressed in tumor cells, have been linked to deregulation of cell proliferation with increase of ROS that, in turn, cause DNA damage [49]. In fact, both Ras and Myc induce metabolic reprogramming of cancer cells with increased glucose and glutamine metabolism and, consequently, increased proliferation and ROS production. In this case, ROS species are represented by superoxide that accumulates after Ras and Myc overexpression. However, the precise mechanism through which Ras and Myc induce ROS increase is still unknown and does not depend on mitochondrial superoxide production [50]. Another important family of transcription factors linked to Ras is represented by the STAT family that is inactivated by increased ROS through oxidation of cysteine residues [51]. Alternatively, increased STAT3 and 5 determine a decrease in mitochondrial ROS production [51]. Interestingly, malignant transformation of mouse embryo fibroblasts by activated Ras oncogene also requires mitochondrial STAT3 and decreased ROS accumulation [52].

## 4. ROS and Hypoxia: Tumor Necrosis and Adaptation

**Figure 3:**
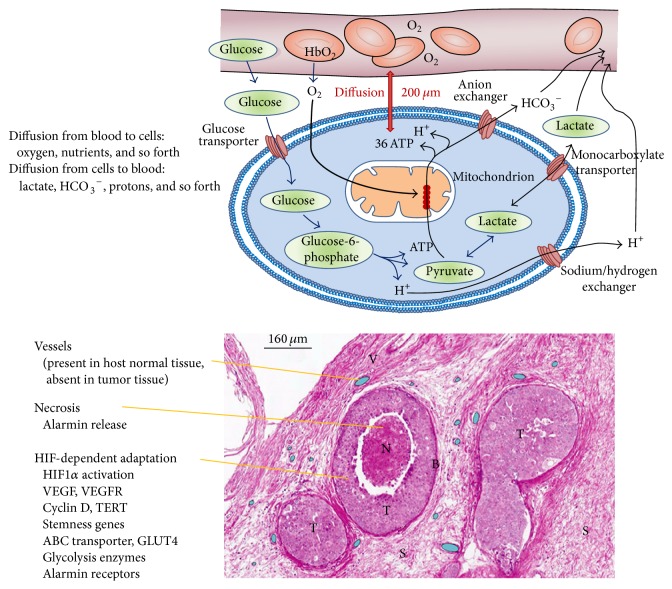
Hypoxia in cancer cells. Exchanges between blood in the vessels and cells are limited by distance and diffusion rate. Oxygen, glucose, and other nutrients diffuse from blood to feed the cells. Lactate, protons, carbonate, CO_2_, and catabolites reach the blood for their disposal. In the early tumor growth, in the absence of angiogenesis, the central regions of tumoral mass, more distant from vessels, undergo necrosis, while peripheral regions survive and adapt to the hypoxia thanks to the HIF-dependent gene expression. Cancer stem cells seem to adapt more easily than differentiated cancer cells [53].

Initial growth of transformed cells, leading to the initial tumor mass, occurs in the absence of or with inefficient angiogenesis. When tumor diameter and the intercapillary distances reach 200 *μ*m (which is the diffusion limit of the oxygen from blood) the tumor tissue becomes hypoxic, with important effects for the tumor microenvironment and for the metabolism of transformed cell itself that becomes more glicolytic [53] (Figure 3). In order to better understand the characteristics of the tumor microenvironment, it is important to consider that average oxygen partial pressure (pO_2_) of tumors, measured by a polarographic pO_2_ sensor, is about 8–10 mmHg or 1.1–1.3%. By contrast, pO_2_ in various human tissues has an average of 35 mmHg or 4.6% [54]. Therefore, it is now a consolidated fact that hypoxia is a characteristic of solid tumors and represents a negative prognostic indicator [54].

As previously described, during this phase, a third prominent role of ROS is evident: hypoxia produces ROS which activate HIF1*α*, by inactivating its inhibitor, PHD (Prolyl Hydroxylase Domain) [53]. Even though this review is focused on the interplay among ROS, HIF1*α*, and NF*κ*B, it is important to keep in mind that hypoxia-driven ROS activates also other transcription factors such as NRF2. NRF2 has an important role in regulating transcription of proteins involved in antioxidant defense thereby reducing ROS accumulation [55]. Importantly, NRF2 and HIF1*α* may act together or independently in regulating, for example, HO-1 expression. Moreover, HIF1*α* regulates NRF2 in some colorectal cell lines [55], whereas silencing NRF2 expression results in HIF1*α* and VEGF reduction indicating a complex and yet unraveled network between these players [55]. In addition, the situation gets complicated by the observation that many human cancers show a significant upregulation of NRF2 correlating with a poor prognosis [55]. For an exhaustive description of NRF2 function in physiological and pathological conditions, we remand the reader to the recent review by Moon and Giaccia [56].

A further ROS increase causes DNA double strand breaks with increase in mutations (genomic instability) and cell damage (lipoperoxidation) leading to* necrosis* of cells that are more distant from vessels. However, the* activation of HIF1α* by ROS in sublethally damaged tumor cells closer to the vessels allows the expression of HIF1*α*-driven genes that contribute to their survival and growth thereby increasing their commitment to malignancy.

*Necrotic damage *includes plasma membrane fragmentation and release of intracellular molecules, some of which constitute alarmins or DAMPs (Damage-Associated Molecular Patterns) [57]. The interaction of released alarmins with their receptors triggers a proinflammatory gene expression in various cell types: resident innate immunity cells or leukocytes, usually expressing a number of alarmin receptors [58]. Importantly, tumor cells may also express alarmin receptors following hypoxia and HIF1*α* activation. Alarmin receptor signaling leads to the activation of NF*κ*B and then to the proinflammatory gene expression. This proinflammatory microenvironment can contribute to tumor progression (see below).

*Activation of HIF1α* leads to the expression of hundreds of genes. Some important HIF1*α*-dependent genes with their role in cancer cell as well as the effect of ROS are reported in Table 2. Many of these genes provide a first impulse (commitment) toward tumor progression. For example, VEGFs and their receptors are responsible for neoangiogenesis and for the possibility to grow above the limit of 400 microns in diameter [59]. Telomerase activation increases the proliferative potential [60]. Finally, changes in intermediate and energy metabolism provide a growth advantage to tumor cells that can quickly use glucose and glutamine [61, 62].

## 5. ROS, HIF1**α**, and HIF1**α**-Dependent Genes

ROS produced during hypoxia have a central role in stabilizing and activating HIF1*α* which in turn triggers the molecular mechanisms important, for instance, to sustain survival, growth, motility, metastasis, and metabolic changes of a transformed cell. However, in some cases, ROS can also directly influence the activity of a number of gene families playing a critical role in pushing a transformed cell toward the acquisition of many hallmarks of malignancy.

### 5.1. ROS and VEGFs and VEGFRs

Increased expression of VEGFs and their receptors VEGF-R1 and R2 is due to the activation of HIF1*α* by ROS and has the fundamental role of activating a tumor-specific neoangiogenesis, allowing the early tumor to grow over the dimensions (200–300 *μ*m), imposed by the simple diffusion of oxygen and nutrients [63]. Alternatively, ROS can also activate the MAPK pathway leading, again, to the increased expression of VEGF [64]. Interestingly VEGF, VEGF-R1, and R2 are expressed in human colorectal samples as well as in human colon cancer cell line, whereas no expression is observed in human normal colonic cell lines. This suggests that VEGF can be produced and secreted by cancer cells to sustain their proliferation and migration. Accordingly, VEGF silencing in colon cancer cells resulted in decreased growth and motility of colon cancer cells [65].

### 5.2. ROS and Telomerase

There are indications about a direct role of ROS on telomerase activity in hepatocellular carcinoma [66]. However, it is believed that the role of ROS may depend on their amount in the cells with low or mid levels being able to activate and high levels to inhibit telomerase activity [67]. Moreover, the effect of ROS on telomerase activity may depend on HIF1*α* as previously demonstrated [68]. Recently, a role of telomerase in regulating cell survival, signaling, and mitochondrial function has been also proposed [69].

### 5.3. ROS and Proliferation

A further contribution to the proliferative potential is given by the HIF1*α*-dependent activation of typical proproliferative genes such as c-Myc and cyclin D1 [70]. As discussed below, the increased proliferation of tumor cells, in which HIF1*α* is active, is also linked to the metabolic reprogramming of these cells.

### 5.4. ROS and Stem Cell Maintenance and Reprogramming

In addition, HIF1*α* activates OCT4 and Notch facilitating stem cell renewal, contributing to the immortalization and increasing survival of cancer stem cells [71, 72]. These observations derive from studies conducted using hematopoietic stem cells (HSC). In fact, HSC pool is present in hypoxic regions of the bone marrow and shows a high expression of HIF1*α* that is essential to maintain stem cell cycle quiescence through a mechanism involving p16/p19 proteins [73]. Moreover, SOX2 and KLF4 can also be activated along with ROS accumulation in glioblastoma cells thereby increasing the number of stem cells [74]. The observation that the canonical stemness genes are all overexpressed suggests the possibility that reprogramming differentiated tumor cells can have a role in increasing and maintaining the tumor stem cell compartment.

### 5.5. ROS and Resistance to Chemotherapy

Resistance of tumor cells and particularly of cancer stem cells is achieved by overexpression of ABC transporters driven by the HIF1*α* transcription factor activated by reduced oxygen tension and/or ROS in the tumor microenvironment [75]. In particular, HIF1*α* has been shown to increase expression of MDR1 [76]. Interestingly, also in this case, the level of ROS achieved inside tumor cells plays an important role. In fact, high levels have been shown to reduced HIF1*α* and MDR expression and survival in spheroids from prostate tumor cells [77].

### 5.6. ROS and Changes in Tumor Metabolism

ROS and HIF1*α* activation are responsible for the large metabolic reprogramming of cancer cells that requires other transcription factors such as Myc and proceeds with the overexpression of proteins such as glucose transporter 1 (GLUT1) for glucose uptake, glutaminase for glutamine usage, hexokinase II (HKII) for glycolysis, and carbonic anhydrase IX (CAIX) for control of intracellular pH, which assures the glucose and glutamine dependency and the fast growth of tumors [61, 78].

### 5.7. ROS Contribution to Invasion and Metastasis

ROS increase in tumor cells contributes to the activation of proteases involved in the recognition and degradation of basement membrane as well as in the formation of invadopodia [79]. Importantly, most of the invasion and metastasis genes are cocontrolled by HIF1*α* and by NF*κ*B, as also discussed in the next paragraph. In particular, ROS activates intracellular signaling mechanisms involving MAPK that depend on NF*κ*B and are upstream of MMPs [79]. Moreover, ROS also activates the recruitment of a series of actin-associated proteins such as cofilin and fascin as well as adhesion (integrins) and signaling (c-Src tyrosine kinases) proteins that assemble together to form the invadopodia [79]. Therefore, the metastatic potential of transformed tumor cells can be increased following upregulation of ROS production.

### 5.8. ROS and Receptors for Alarmins

In the presence of a hypoxic environment a number of cell types, including cancer and normal stem cells, express* de novo* or overexpress different alarmin receptors (similar to those present in activated leukocytes or CD45^+^ cells) [80, 81]. RAGES, P2X7, TLRs, and others, upon activation by alarmins released by necrotic cells, converge in the activation of NF*κ*B with a robust proinflammatory gene expression. This represents the key event to bridge the hypoxia to the adaptation with the expression of hundreds of genes related to the IRR and, very importantly, to the acquisition of classical properties of the malignant phenotype. This picture includes also the so-called EMT (epithelial-mesenchymal transition), in which involved genes can be HIF1*α*- and/or NF*κ*B-dependent target [82].

## 6. ROS, NF***κ***B Activation, and the Full Acquisition of Hallmarks of Malignancy

The inflammatory response is finalized to defend cells by eliminating or detoxifying the harmful agents and to repair cell/tissue damage through differentiation of resident or recruited stem cells or by forming a scar of connective tissue. ROS are important players in both defensive and repairing functions of the inflammatory-reparative response (IRR). However, ROS can also cause cell damage, depending on the type, on the local concentration, and on how long and how specifically they interact with cell components [38, 83].

In the classical (“physiological”) IRR, defense and repair are efficiently coordinated by NF*κ*B [84]. This transcription factor becomes fully activated through many synergic and confirming signals, such as cytosolic ROS, exogenous alarmins (i.e., virus, bacteria, other parasites, crystals, and fibers), and endogenous alarmins released by damaged and necrotic cells [85]. This signaling leads to NF*κ*B nuclear translocation and activation and expression of ROS producing enzymes such as NADPH-oxidases, COX2, iNOS, and 5-lipoxygenases [86].

Once NF*κ*B has been activated, a complex gene response occurs, with the expression of genes belonging to specific gene families including a large number of members functionally related to the inflammatory and reparative response (see Table 3). Individually most of these genes have been implicated in the acquisition of crucial properties of the malignant phenotype, providing a coherent theoretical framework to explain the acquisition of most of the malignant hallmarks as an integrated response and adaptation to the tumor environment.

### 6.1. ROS and Inducible Enzymes (NOX, COX2, 5-LOX, and iNOS)

Inducible enzymes produced in activated leukocytes upon activation of NF*κ*B are responsible for the production of mediators such as prostaglandins, leukotrienes, plasmalogens, and NO, leading to the manifestation and amplification of the IRR. Their presence in tumor microenvironment and their expression by tumor cells have been two of the earliest observations involving inflammation in the pathogenesis and progression of cancer [87]. Molecules produced by these enzymes contribute to many aspects of tumor progression such as neoangiogenesis, recruitment of leukocyte to the tumor microenvironment, and changes for EMT [88]. Almost 15 years ago a landmark epidemiological study suggested that the use of low-dose aspirin for cardiovascular prevention drastically reduced the risk for colon cancer [89]. These epidemiological observations stimulated a number of other retrospective studies and controlled clinical trials on aspirin and other COX2 inhibitors in preventing tumors and their progression, giving rise to a new era in understanding the role of inflammation in tumor pathogenesis.

### 6.2. ROS, Cytokines, and Their Receptors

ROS have been shown to induce cytokine synthesis in different systems either directly or following activation of NF*κ*B [90, 91]. Cytokines have a direct influence on IRR by targeting leukocytes, by polarizing the response as Th1 or Th2 and by stimulating the proliferation of target cells (CD45^+^) to reinforce and amplify the IRR [92]. Cytokines are present in most human tumor microenvironment, being produced by cancer cells themselves and/or by leukocyte infiltrate [93, 94]. Interestingly, tumor cells also express receptors for various cytokines in parallel with their degree of malignancy [92]. Therefore, thanks to the presence of cytokine receptors, tumor cells can be strongly influenced in their biology, such as proliferation rate (IL-2), and in their polarization (Th1 cytokines) and, probably, in the expression of adhesion molecules and their counterreceptors, thus influencing the homing for metastasis [95, 96].

### 6.3. ROS and MMPs and TIMPs

Matrix metalloproteinases (MMPs) and tissue inhibitor of metalloproteinases (TIMPs) are HIF1*α*- and NF*κ*B-dependent genes normally expressed in activated leukocytes. ROS can activate MMP synthesis either directly [97] or, more frequently, through NF*κ*B [98, 99]. It is well known that disruption of the MMP/TIMP activity ratio with a gain-of-function of proteasic activity over basement membrane and extracellular matrix proteins is present in malignant tumors and parallels the invasive potential [100]. Therefore, the key event for demolishing the physiological tissue barrier and starting invasion is basically controlled by both HIF1*α* and NF*κ*B through the expression of these genes.

### 6.4. ROS, Adhesion Molecules, and Their Counterreceptors

The activation of NF*κ*B in leukocytes finely reprograms the expression of adhesion molecules for migration and for homing at constitutive district tissue or at damaged site. A ROS-induced NF*κ*B-dependent and/or cytokine-dependent new expression of adhesive molecules occurs also in tumor cells, allowing for a number of biological changes typically related with malignancy [101, 102]. These changes include the ability to detach from the original tissue (i.e., cadherins), the ability to migrate following a specific chemotactic gradient and a path of ECM molecules (receptors for chemokines and integrins), and, finally, the identification of the homing site represented by activated endothelial or other tissue cells (ICAM-1, selectins, and their counterreceptors) [103].

### 6.5. ROS, Chemokines, and Their Receptors

Tumor cells express both chemokines and their receptors in parallel with their degree of malignancy [104]. The production of chemokines gives rise to a gradient which is probably the main responsible for the attraction of leukocytes and mononuclear infiltration in advanced tumors [104]. More importantly, the expression of chemokine receptors is a crucial event for the occurrence of metastasis. In fact, detachment from the primary tumor tissue must be followed by a vectorial migration along a chemotactic gradient, which implies the presence of specific receptors for the chemoattractant. CXCR4, a receptor for SDF1*α*, is the best characterized receptor in tumor cells and has been definitely associated with progression and prediction of metastasis in many human tumors [104]. Interestingly, ROS can enhance CXCR4 function in prostate cancer cells [105]. Moreover, chemokines and their receptors are under the control of NF*κ*B and can be, therefore, induced by ROS.

### 6.6. ROS, VEGFs, and VEGFRs

As also described above when talking about the role of ROS in inducing HIF1*α* and VEGF, in order to be clinically relevant and detectable by the present imaging techniques, a tumor needs to grow at the dimension of a few mm in diameter. At the same time, this tumor must activate a process of neoangiogenesis, with an adequate expression of VEGFs and VEGFRs. VEGFs can be produced by activated leukocytes and mesenchymal cells present in the tumor microenvironment or, more importantly, by tumor cells themselves under the influence of activated HIF1*α* and NF*κ*B [63, 106]. In the last case, it has been demonstrated that cancer cells (probably tumor stem cells and progenitors) may express also VEGFRs, suggesting the possibility that tumor cells can contribute to the formation of their new vascular tree [65].

### 6.7. ROS, Growth, and Survival Factors

HIF1*α* and NF*κ*B control a number of growth and survival factors and their receptors. This has been demonstrated in activated leukocytes (involved in tissue repair) and in hypoxia activated tumor cells. This is an additional advantage for tumor growth and a prerequisite for the establishment of a secondary metastatic tumor. The “seed and soil” hypothesis predicts that a favorable tissue environment is relevant for the occurrence of a metastasis [107]. In this case, growth and survival factors can be provided by activated leukocytes or mesenchymal cells of the microenvironment and by tumor cells themselves in which proliferative pathways are already activated (transforming oncogenes) or in which these genes are overexpressed upon ROS-dependent NF*κ*B activation [108].

### 6.8. ROS and Acute-Phase Proteins

Acute-phase proteins have been considered as plasma markers useful to evaluate the systemic IRR. They include soluble and cell bound isoforms, such as C reactive protein, pentraxin-3, and other pentraxins; their functions are only partially elucidated. Similar to the other NF*κ*B-dependent genes, they are expressed or overexpressed in hypoxia-activated tumor cells and in activated leukocytes. Their function in tumor progression is still debated. On one hand, they seem to inhibit tumor cell proliferation and to decrease with progression [109]; on the other hand they can be highly expressed in malignant cells compared to the host normal tissue [110–113].

### 6.9. ROS, SOCS, and Negative Regulators

NF*κ*B activation includes also the expression of a number of proteins that function as negative key-regulator of IRR, such as SOCS-1 [114]. This latter protein is a member of SOCS family that suppresses the cytokine signaling via JAK/STAT, downregulates TLR expression and signaling, and decreases NF*κ*B activity and duration [115]. This family and other negative regulators are considered as part of the normal feedback control of the IRR. As predicted by our hypothesis, SOCS-1 decreased in hypoxia-activated cells, as a physiological response of HIF1*α*-NF*κ*B integrated activation [116].

## 7. ROS and Sirtuins in Modulating Cell Redox Status and HIF1***α***/NF***κ***B Pathway

In mammals there are seven Sir2 homologs (SIRTs 1–7). Sirtuins are either class III nicotinamide adenine dinucleotide- (NAD^+^-) dependent deacetylase, desuccinylase, demalonylase, deglutarylase, or ADP-ribosyltransferases [117, 118]. Their dependence on NAD^+^ directly links Sirtuins activity to the metabolic state of the cells and to ROS. For this reason, Sirtuins have been implicated in many physiological functions such as gene silencing, cell death, longevity, inflammation, cancer and, importantly, the regulation of ROS levels through both ROS production and detoxification [117]. In addition, Sirtuins deacetylate and then directly regulate the activity of both HIF1*α* and NF*κ*B. However, while only for SIRT1, 2, 3, and 6 this regulatory function has been clearly demonstrated, it is now clear that also the other Sirtuins influence a number of metabolic pathways, converging in ROS regulation.

### 7.1. SIRT1

SIRT1 can be a target of damaging ROS and this may cause its relocalization, inactivation, and degradation. In particular, ROS can oxidize SIRT1 cysteine residues thereby inhibiting its activity and targeting the protein towards proteasomal degradation [119]. In fact, oxidative stress associated with inflammation downregulates the expression and the activity of SIRT1 and [119, 120] SIRT1 can be cleaved in inflammatory conditions [121, 122].

Another mechanism through which ROS can reduce SIRT1 activity involves NAD^+^. In fact, oxidative stress reduces the cellular level of NAD^+^ suppressing the SIRT1-mediated signaling [123]. Interestingly, the increase of oxidative stress observed during aging in several rat tissues is accompanied by a concurrent decrease in the level of NAD^+^ and in the activity of SIRT1 [124]. Similar changes were observed in human skin [125]. Recently, it was shown that, in mammalian cells, oxidative stress (H_2_O_2_) causes a cytosol to nucleus translocation of SIRT1 followed by its chromatin binding [126]. At least part of the SIRT1 pool appears to be targeted to double strand breaks, where it promotes repair and genomic stability. Genes that are normally silenced by SIRT1 become derepressed, leading to an altered pattern of transcription that resembles that of the aging brain, which is known to be subjected to significant oxidative stress. Finally, oxidative stress also activates PARP-1, which consumes cellular NAD^+^ storage thereby decreasing SIRT1 activity [127].

On the other hand, downstream effects of SIRT1 on various transcription factors can affect directly ROS production and decrease or increase ROS resistance by influencing ROS detoxifying/scavenging systems. Importantly, SIRT1 deacetylates both HIF1*α* and NF*κ*B. In the case of NF*κ*B, SIRT1 has been shown to deacetylate and inactivate the p65/relA component with inhibition of the NF*κ*B complex [128]. In fact, both* in vitro* and* in vivo* observations have shown that SIRT1 or activation of SIRT1 by resveratrol and other polyphenols decreases inflammatory response by deacetylating and inhibiting NF*κ*B [129]. These results are particularly interesting considering the central role of NF*κ*B in many cellular pathways involved, for instance, inflammation, aging, and cancer. Controversial results have been reported, instead, for SIRT1/HIF1*α* signaling. In fact, it is not yet clear if SIRT1 is influenced or not by hypoxia. Some reports indicate that hypoxia increases SIRT1 levels, whereas others indicate that hypoxia decreases SIRT1 [130, 131]. Under hypoxia SIRT1 deacetylates HIF1*α*; however, such reaction in some cases decreases HIF1*α* activity, whereas in others it increases HIF1*α* activity. Obviously, more data must be accumulated on different cell lines, tissue,* in vivo* models, and tumors before the real function of SIRT1 on HIF1*α* can be delineated. Moreover, it is also possible that SIRT1 action of HIF1*α* differs in different tissues and organs. Given the widespread actions of SIRT1 in mammalian cells, it is likely that we have only scratched the surface of how this Sirtuin influences and interacts with ROS.

### 7.2. SIRT2

The connection between SIRT2 and ROS is still at the beginning. However, some results have shown that oxidative stress increases SIRT2 expression and nuclear accumulation. Nuclear SIRT2 then deacetylates and activates DNA binding of Foxo3a transcription factor that, in turn, results in increased transcription of its target genes and finally a decrease of ROS [132]. SIRT2 has also been shown to inhibit ROS production following LPS treatment of macrophages by suppressing NF*κ*B activation [133]. In fact, SIRT2 has been shown to deacetylate subunit p65 of NF*κ*B on lysine 310 (K310) in the cytoplasm [134]. In this way SIRT2 inhibits NF*κ*B activation and transcription of NF*κ*B target genes following TNF stimulation [134]. In fact, SIRT2 silenced cells have an increased activation of NF*κ*B and a lower percentage of cell death following TNF exposure [134]. Finally, addition of a cell permeable PEP-1-SIRT2 protein to murine macrophages resulted in a reduction of ROS due to an increase in antioxidant enzymes such as MnSOD and catalase [135]. The precise role of SIRT2 in tumors is still a matter of debate with some reports showing a correlation between SIRT2 levels and poor prognosis in non-small-cells lung cancer or progression of cervical cancer [136, 137], whereas others report a correlation between low levels of SIRT2 and non-small lung cancer [138]. However, the current literature points to an oncogenic role of SIRT2 since its inhibition results in an impaired growth of lung, cervical, sarcomas, gliomas, and so forth by regulating cell cycle and autophagy [139, 140].

### 7.3. SIRT3

The expression and deacetylating activity of the mitochondrial Sirtuin SIRT3 have been extensively associated with a decrease of oxidative stress and an increase of cell vitality and lifespan. In particular, in arsenic-treated adipocytes, reduction of ROS by SIRT3 is due to the activation of transcription factors such as FOXO3a that, in turn, increases expression of ROS scavenging enzymes [141]. Deacetylation of FOXO3 by SIRT3 decreases proteasomal degradation of the former and increases resistance to ROS [141]. Decrease in ROS production after SIRT3 overexpression or activation (resveratrol) has been documented in different systems and pathologies such as age-related dysfunction of the auditory system [142], doxorubicin toxicity of cardiomyocytes due to oxidative stress [143], and hypoxic stress of endothelial cells [144]. In fact, SIRT3 control of HIF protein stability is achieved by controlling ROS levels as well as other metabolic pathways [145]. In particular, by decreasing ROS levels, SIRT3 stabilizes HIF degrading enzyme Prolyl Hydroxylase (PHD) lowering HIF1*α* levels [146]. Interestingly, SIRT3 deficiency is associated with tumor growth in xenografts and SIRT3 expression is lowered in several cancers and cancer cell lines [146].

### 7.4. SIRT4

Very little is known about this mitochondrial Sirtuin and its role in the regulation of oxidative stress response. However, SIRT4 ADP-ribosylates and inactivates glutamate dehydrogenase 1 (GDH-1) decreasing insulin secretion in pancreatic cells [147]. Interestingly, SIRT4 seems to increase sensitivity of HeLa cells to oxidative agents and such effect has been linked to GDH-1 inhibition [148]. The mechanism involves a SIRT4-dependent opening of the permeability transition pore in the mitochondria with increased cell death following exposure of cells to oxidative stress [148]. Given the fact that SIRT4 is involved in the regulation of mitochondrial metabolism, it has been postulated that this Sirtuin must play an important role during metabolic reprogramming of cancer cells [149]. In particular, SIRT4 has been reported to have a tumor suppressive role because of its ability to suppress glutamine metabolism by ADP-ribosylation and inhibiting GDH [150]. In fact, SIRT4 suppresses Myc-induced B cell lymphoma and survival of human colorectal cancer cells [151, 152].

### 7.5. SIRT5

As in the case of SIRT4, the study of the role of this mitochondrial Sirtuin in oxidative stress response is still at the beginning. One study has shown that SIRT5 desuccynilates and activates Cu/ZnSOD, an effect that is accompanied by a reduction of ROS levels [153]. Other studies have, instead, linked SIRT5 desuccynilating activity to the inhibition of glutamine metabolism that produces glutamate necessary for the production of the antioxidant glutathione [154]. Therefore, in this case, SIRT5 could determine an increase in ROS. Of note, many tumors show a decreased expression of SIRT5 and an increased glutamine metabolism [155, 156]. Moreover, increased glutamine metabolism determines the production and diffusion of ammonia that, in turn, stimulates autophagy that limits ROS and DNA damage and inhibits tumor initiation [157]. However, autophagy has also a central role in the survival of established tumors by removing damaged organelles and toxic agents [158]. It must be concluded that the role of glutamine metabolism, mitochondrial Sirtuins, and ROS depends on the cancer type and, more interestingly, on the stage and context of the tumor.

### 7.6. SIRT6

SIRT6 has been linked to ROS, inflammation, and cancer by several studies. In particular, the expression of this nuclear Sirtuin is reduced in endothelial cells in the presence of ROS with acquisition of a senescent phenotype [159]. On the other hand, SIRT6 deacetylation of histone H3 regulates genes important for metabolism and telomeres maintenance thereby promoting resistance to oxidative stress damage [160]. SIRT6 controls cell metabolism by deacetylating and inactivating transcription factors such as HIF, NF*κ*B, and Myc [161]. In fact, SIRT6 protects cardiomyocytes from hypoxia by increasing Bcl-2 and decreasing NF*κ*B expression [162]. The inhibition of NF*κ*B by SIRT6 determines its control over inflammation. Accordingly, SIRT6 downregulation is followed by an increase of NF*κ*B transcriptional activity and release of inflammatory cytokines such as IL-1*β* or synthesis of COX2, MMPs, and adhesion molecules [163]. Moreover, overexpression of SIRT6 prevented inflammation in a mouse model of collagen-induced arthritis [164]. Finally, SIRT6 has also been linked to malignancy. To this effect, SIRT6 is considered as a tumor suppressor because it deacetylates and inactivates HIF and NF*κ*B but, more importantly, because it regulates the activation of the DNA repair machinery after both double strand breaks (DSB) and base excision repair (BER). In fact, SIRT6 declines with age or SIRT6 downregulation is associated with a decrease of BER [165]. On the other hand, however, the increased lifespan associated with SIRT6 could imply that SIRT6 may promote tumor formation and, in fact, recently an increase of SIRT6 has been associated with enhancement of tumorigenicity of hepatocellular carcinoma cells in the presence of TGF-*β*1, H_2_O_2_, and HOCl [166].

### 7.7. SIRT7

Initially identified as an activator of RNA polymerase I [167], SIRT7 is now also linked to tumor transformation by controlling cellular proliferation and survival. In fact, SIRT7 has been shown to reduce DNA damage markers following doxorubicin treatment of osteosarcoma cells as well as cell cycle arrest markers such as p21. Moreover, SIRT7 decreased apoptosis and p53 response pathway [168]. Furthermore, SIRT7 inactivation suppresses migration of cancer cells and tumor metastasis formation in a mouse model [169]. However, SIRT7, at least in cardiomyocytes, has an important role for cell survival and function because of its ability to deacetylate and inhibit p53, to protect from oxidative stress, and to reduce inflammation. In fact, SIRT7-deficient mice develop cardiac hypertrophy and inflammation and have a shorter lifespan [170].

In conclusion, giving the fact that Sirtuins regulate both HIF1*α* and NF*κ*B and the central role that these two transcription factors have during tumor progression, the possibility to act on Sirtuins in order to control HIF1*α* and NF*κ*B has drawn much attention. Therefore, presently, great deals of efforts have been put in producing Sirtuins modulators. Several natural compounds such as resveratrol, quercetin, piceatannol, and other polyphenols have been shown to modulate Sirtuins function and particularly SIRT1 [171, 172]. However, their action is not limited to SIRT1 but influences other enzymes such as phosphodiesterases (PDEs) and AMP kinase (AMPK) [173]. Unfortunately, so far, no specific inhibitors or activators for other Sirtuins are available.

## 8. Conclusions

**Figure 4:**
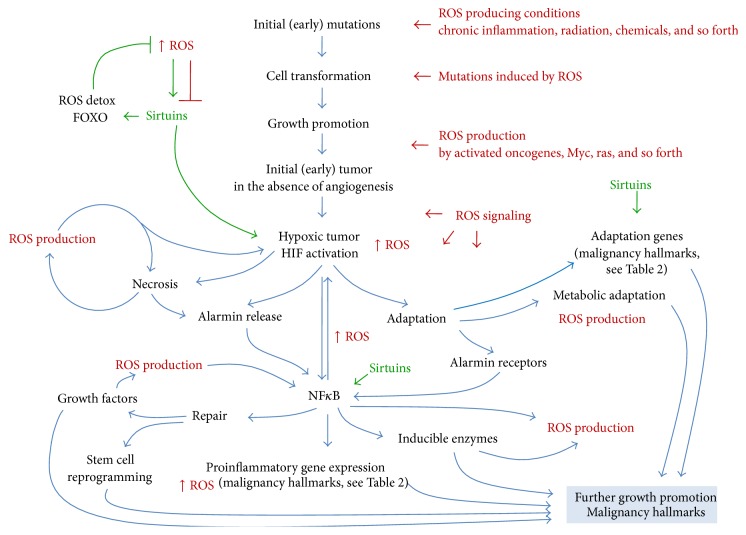
The natural history of a tumor from initial cell transformation to progression occurs and develops in a ROS-rich milieu which deeply influences, reinforces, and amplifies the different steps of the progression, the role of various molecular players, especially DNA repairing mechanisms, HIF, NF*κ*B, and Sirtuins, and the full acquisition of all hallmarks of malignant phenotype.

This review has been an occasion to summarize evidences that cell redox status is the milieu where many players can contribute* initially* to the cell transformation and* successively *to the progression of the malignancy. Initially there is the formation of early small tumors in the absence of angiogenesis which then progress to grow as a vascularized clinically evident tumor, with the acquisition of all the hallmarks of malignant phenotype. From a molecular point of view, two transcription factors, namely, HIF1*α* and NF*κ*B, may be considered as master regulators of tumor cell adaptation to ROS. In fact, both HIF1*α* and NF*κ*B are induced by ROS and, in turn, can regulate ROS production to sustain tumor cell survival and growth. An overview of the different aspects discussed in this review is summarized in Figure 4 in which ROS production and signaling as well as ROS effect on tumor cell metabolism and behavior are indicated. Figure 4 also indicates the important role of hypoxia and transcription factors HIF1*α* and NF*κ*B in orchestrating the tumor cell response to ROS. Therefore, it is conceivable that a number of exogenous agents and strategies aimed at influencing their activity could be used to reduce tumor transformation and progression.

## Figures and Tables

**Table 1:** Classes of ROS and their properties.

Radical	Structure	Reactivity	Half-life	Production/localization	Diffusion	Targets	Biological effect	Pathological effect
Hydroxyl radical	OH^•^	High	10^−9^ sec	Mitochondria Phagosome Endoplasmic reticulum (ER)	Highly localized where is produced	Any cell component	Unknown	Toxicity

Superoxide	O_2_^−^	Low	1–15 minutes	Mitochondria cytosol ER Peroxisome	Localized, it can diffuse through an anion channel	Fe-S centers Nitric oxide	Protein modification (activation or inhibition)	Protein damage

Hydrogen peroxide	H_2_O_2_	Moderate Reversible	Hours to days	Mitochondria cytosol ER Peroxisome	Diffuse, it can travel through aquaporins	Iron-sulphur Cysteine residues	Activation of signaling	Mutation, accumulation, and genomic instability

**Table 2:** HIF-dependent genes in hypoxia adaptation in determining malignancy hallmarks.

HIF-dependent genes	Adaptation phenotype	ROS effect	References
VEGFs and VEGFRs	Neoangiogenesis, repair	Indirect	[63–65]
TERT (telomerase)	↑ telomere length and proliferative potential	Direct and indirect	[66–69]
Cyclin D1, cyclin D2	Increased proliferation	Indirect	[70]
TERT; c-Myc, SOX2, OCT4, KLF4, Notch	Stem cell renewal, differentiated cell reprogramming	Indirect	[71, 72]
ABC transporter	Drug resistance	Indirect	[75–77]
ALDA, PGK, GLUT-1	Changes in energy metabolism	Indirect	[61, 78]
PDGF, chemokine receptors	Motility and polarized migration	Indirect	[104, 105]
MMP9, MMPs	Integrity of basement membrane; invasiveness	Direct and indirect	[97–100]
Alarmin (DAMPs) receptors	NF*κ*B activation; IRR gene express	Indirect	[80–82]

**Table 3:** NF*κ*B-dependent genes and their role in tumorigenesis.

NF*κ*B-dependent gene families	Proinflammatory phenotype and malignancy hallmark	ROS effect	References
Inducible enzymes	Vasodilatation, migration	Indirect	[87–89]
Cytokines and receptors	Local amplification of IRR	Direct and indirect	[90–94]
MMPs and TIMPs	Invasion, migration	Direct and indirect	[97–100]
Adhesion molecules and their counterreceptors	Detachment, homing	Indirect	[101–103]
Chemokines and receptors	Migration, homing	Direct and indirect	[104, 105]
VEGFs and VEGFRs	Angiogenesis, repair	Indirect	[63–65, 106]
Growth and survival factors	Proliferation, antiapoptosis, repair	Indirect	[107, 108]
Acute-phase proteins	IRR amplification, chemotaxis, repair	Indirect	[109–113]
SOCS-1	Negative regulator of IRR	Indirect	[114–116]
